# Comparison on radiation effective dose and image quality of right coronary artery on prospective ECG‐gated method between 320 row CT and 2nd generation (128‐slice) dual source CT

**DOI:** 10.1002/acm2.12911

**Published:** 2020-06-08

**Authors:** Ren‐Feng Li, Chang‐Long Hou, Huang Zhou, Yan‐Shan Dai, Li‐Qin Jin, Qian Xi, Jian‐Hua Zhang

**Affiliations:** ^1^ Department of Radiology Shanghai East Hospital Tongji University School of Medicine Shanghai China

**Keywords:** 2nd generation dual source CT scan, 320 row CT scan, heart rate, image quality score, radiation dose

## Abstract

This retrospective study was to compare the image quality of right coronary artery (RCA) and effective radiation dose on prospective ECG‐gated method between 320 row computed tomography (CT) and 2nd generation (128‐slice) dual source CT. A total of 215 candidates underwent CT coronary angiography using prospective ECG‐gated method, 120 patients enrolled in 320 row CT group, and 95 patients in dual source CT group. We divided RCA image quality scores as 1/2/3/4, which means excellent/good/adequate/not assessable and heart rates were considered, as well as the radiation dose. There is no statistically significant difference of RCA image quality of Score 1/2 between 320 row CT and 2nd generation dual source CT, but lower heart rate (<70/min) improved RCA image quality. Meanwhile, the 2nd generation dual source CT scan have significant lower radiation dose. For patients with high level heart rate variation, both prospective ECG‐gated method of 320 row CT scan (Toshiba) and 2nd generation dual source CT scan (Siemens) basically provided good image quality on RCA. There is an advantage of effective radiation dose reduction in prospective ECG‐gated method using the 2nd generation dual source CT scan. After the iodine contrast agent was injected into elbow vein, the threshold triggering method was used to carry out prospective gated scanning, and the acquired fault image was reconstructed by the standard post‐processing software of each manufacturer. The radiation dose value is obtained through the dose report automatically generated after each scan.

## 1 INTRODUCTION

Coronary atherosclerotic heart disease is the leading cause of death worldwide.^1^ Many trials to date have investigated the diagnostic accuracy of coronary computed tomography angiography (CCTA) when compared to the gold standard diagnostic test, invasive coronary angiography.^1^ Until now, CCTA is one of the important achievements on CT technology in the past decade, which provides a convenient method for the diagnosis of coronary artery disease.^2^ Coronary computed tomography angiography was proved to have a high negative predictive value. Coronary computed tomography angiography plays an important role as a less invasive investigation of coronary atherosclerotic heart (CAD) disease proved by its high negative predictive value, which also decreases the necessity of invasive digital subtraction angiography (DSA) for CAD diagnosis.^3^

Nowadays, the diagnostic performance of coronary CT angiography has been significantly improved with the technological developments in multislice CT scanners from the early generation of 4‐slice CT to the latest 320‐slice CT scanners.^4^ Different CT manufactures has continually announced and recommend their new models with technical advantages on higher image quality, faster scanning time, wider detector range, and lower effective radiation dose. With the increase in the width of the practical application of the detector, the width of the collimation in front of the spherical tube is correspondingly increased, and the eave effect and the invalid scanning radiation dose at the edge of the scanning range are also increased. Despite its ongoing success and worldwide clinical implementation, it remains an often‐challenging procedure in which image quality, and hence diagnostic value, is determined by both technical and patient‐related factors.^5^ Two examples of scanning models are 320 row CT [320‐detector row dynamic volume computed tomography, (DVCT)] (Toshiba) and 2nd generation (128‐ slice) dual source CT scan (Siemens), and prospective electrocardiographic (ECG)‐gated method is what we selected to study. Subtraction CCTA using a second‐generation 320‐detector row CT showed improvement in diagnostic accuracy at segment base analysis in patients with severe calcifications.^6^ Low‐dose CT is highly effective and can reduce the potential risk of exposure to ionizing radiation.^7^, ^8^ Compared with retrospective ECG‐gated method, prospective ECG‐gated protocol has the advantage of low effective CT radiation dose exposure, and it is the method that uses forward‐looking prediction of R wave timing, step‐and‐shoot axial acquisition with no table motion while imaging, and for single type cone beam reconstruction.^9^ To the best of our knowledge, since prospective cardiac‐gated CCTA was always performed with Toshiba 320‐detector or Siemens 2nd generation 128‐slice dual‐source scanners,^10^ there is no comprehensive study comparing these two specific CT models of different manufactures, based on their image quality on right coronary artery (RCA) and effective radiation dose reduction. In this retrospective study, we share our experience comparing the corresponding parameters on these two mentioned CT devices, discuss their corresponding technologies and explore which device has better image quality and lower radiation dose.

## 2 MATERIALS AND METHODS

### 2.A Scanning method

Using double‐syringe injector, 60 ml contrast agents and 30 ml saline were injected through cubital vein by 5.0 ml/s. The injection was triggered by corresponding threshold values of CT devices (threshold values of 320 row CT (Toshiba) and 2nd generation (128‐slice) dual source CT (Siemens) were 150 and 120 HU, respectively), the regions of interest (ROI) were located in the aortic root. The 320 row CT (Toshiba) was only operated in prospective gated scan with the scan parameters: tube voltage at 100 kV with automated tube current modulation and slice thickness at 0.5 mm × 320. There is a range of 16 cm scanned in a single gantry rotation. Acquisition phase of cardiac cycle for heart rate (HR) below 70/min and the selection of R‐R interval is at about 50%–80%. When HR is above 70/min and the selection of R‐R interval, acquisition phase is at 30%–55%. The image reconstruction was using iterative reconstruction (IR) process. The scanning of 2nd generation (128‐slice) dual source CT (Siemens) is using step‐ and‐ shot method with setting of step‐by‐step distance at 38.4 mm. X ray tube voltage is at 80–100 kV with auto milliampere second. Acquisition phase of cardiac cycle for heart rate (HR) below 75/min and the selection of R‐R interval is at about 30–50%. When HR is above 75/min and the selection of R‐R interval, acquisition phase is at 50–80%. The corresponding image reconstruction is using iterative reconstruction (IR) method.^11^ (scan parameters is shown in Table 1).

**Table 1:** Scan parameters.

	Tube voltage (Kv)	Tube current	Slice thickness	Collecting R‐R interval phase	Scanning mode	Reconstruction mode	Trigger scan threshold (HU)	Contrast dose (ml)	Contrast injection rate (ml/s)
320 row CT	100	Automatic	0.5 mm × 320	HR < 70/min: 50–80%	Prospective gated scanning	Iterative reconstruction	150	60	5
				90/min > HR ≥ 70/min: 30–55%					
2nd generation (128‐slice) dual source CT	80–100	Automatic	0.625 mm × 128	HR < 75/min: 30–50%	Prospective gated scanning	Iterative reconstruction	120	60	5
				90/min> HR≥75: 50‐80%					

### 2.B Image post processing

320 row CT scan (Toshiba) and of 2nd generation (128‐slice) dual source CT scan (Siemens) default iterative reconstruction IR method so as to obtain four different images: (a): maximum intensity projection (MIP) to investigate the relationship between vascular calcification; (b): curved planar reformations (CPR) to observe the stenosis of single vessel; (c): volume rendering technique (VRT) and multiplanar reformations (MPR) to observe the relationship between coronary arteries and myometrium.

### 2.C Clinical data

Candidates were retrospectively selected from January 2015 to August 2015, all of them underwent coronary CT angiography (CCTA) using the 320 row CT scan (Toshiba) or 2nd generation (128‐slice) dual source CT scan (Siemens), respectively. There were 215 candidates (male: 110; female: 105) and range of age at 32 to 86 yr old (average: 59 y/o). Among, 120 candidates received 320 row CT scan (Toshiba) and 95 candidates received 2nd generation (128‐slice) dual source CT scan (Siemens). During CCTA examination, there were 110 candidates with HR <70/min, and 105 candidates with HR 70–90/min. All patients were randomly assigned to use the two devices for CCTA. Among the candidates with uncontrollable arrhythmia (with HR difference >10/min), atrial fibrillation, and inadequate breath holding, which used retrospective ECG‐gated method were excluded. All patients were treated with sublingual nitroglycerin 0.5 mg at 5 min before the CT examinations. For the candidates with HR > 90/min, they used 50 mg beta‐blocker to stabilize the heart rate at 30–60 min before CT scan. After beta‐blocker controlled, if the candidates still not able of achieving the desired heart rate (<90/min), still persistent arrhythmia with HR difference >10 beats/min or still atiral fibrillation, they underwent retrospective gated method by 2nd generation (128‐slice) dual source CT scan (Siemens). These candidates were excluded from this study. Those could not hold breath were also excluded. Only the cases using prospective gated method of scanning on the two devices for CCTA, were included in this study. This study was conducted in accordance with the declaration of Helsinki. This study was conducted with approval from the Ethics Committee of Tongji University School of Medicine. Written informed consent was obtained from all participants.

### 2.D Quality assessment of right coronary artery image

**Fig. 1:**
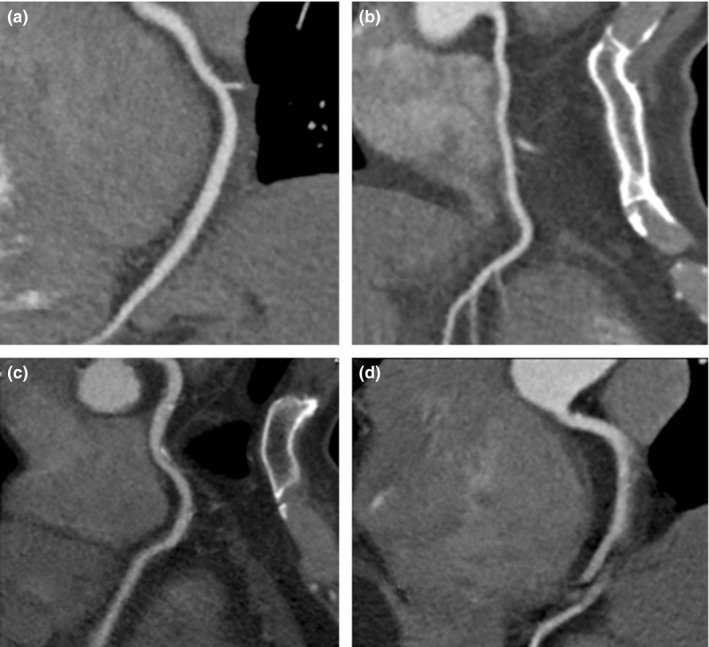
Different image quality of right coronary artery (a: Score 1, b: score 2, c: score 3; d: score 4).

Right coronary artery (RCA) emerges from the thoracic aorta into the atrioventricular groove. It descends through the groove, then curves posteriorly, makes a bend at the crux of the heart and continues downward in the posterior interventricular sulcus. Since contraction of atrial and ventricular are not synchronous, it leads to a great possibility of motion artifacts for CCTA. A good image quality of RCA is most representative to act as an assessment on the high performance of CCTA system. The exclusion criteria for candidates are: (a) diameter of RCA calibre <1.5 mm, (b) stent placement; (c) severe calcified plaques (total calcium score > 400). Assessment of image quality of RCA was performed individually by two experienced radiologists on CCTA. They would dicuss the inconsistent results. And then finally reach the final results. The grading of image assessment on good or worse image quality of RCA is divided as both readers semi‐quantitatively assessed the image quality of each coronary segment on a four‐point ranking scale (Fig. 1):^12^ (a) excellent (low image noise, clear boundary of coronary artery lumen, no artifact, excellent image quality) [Fig. 1(a)]; (b) good (low image noise, slightly fuzzy or mild artifact of coronary artery lumen boundary, image quality meeting the diagnosis) [Fig. 1(b)]; (c) adequate (high image noise, fuzzy or moderate artifact of coronary artery lumen boundary, image quality having diagnostic value Influence) [Fig. 1(c)], and (d) not assessable (image noise is large, the boundary of coronary artery lumen is unclear or serious artifact, the continuity of blood vessel is interrupted, and the image quality cannot meet the diagnosis) [Fig. 1(d)].

### 2.E Radiation dose assessment

Radiation dose reports on the two CT devices were generated automatically by the corresponding software from CT scanning equipment. By using radiation dose index (CTDI vol) and dose length product (DLP), multiplied by DLP and k conversion coefficient. (k), to estimate the effective dose (ED). The k value is based on adult chest for estimation, k = 0.014 mSv mGy^−1^ cm^−1^.^13^

### 2.F Statistical analysis

Statistical work was performed using the SPSS17.0 software (SPSS Inc., Chicago, IL, USA). Those 215 cases of patients are divided into two groups according to the different heart rates (HR <70/min; HR70–90/min). In each of the two groups, further matching and analysis was done to prospective ECG‐gated scan method between 320‐row CT scan (Toshiba) and 2nd generation (128‐slice) dual source CT scan (Siemens). Independent *t*‐test on samples was performed to obtain mean and standard deviations for the right coronary image grading of quality and the effective radiation dose. Statistically significant difference is defined if *P* < 0.05.

## 3 RESULTS

### 3.A Image quality score on RCA

**Fig. 2:**
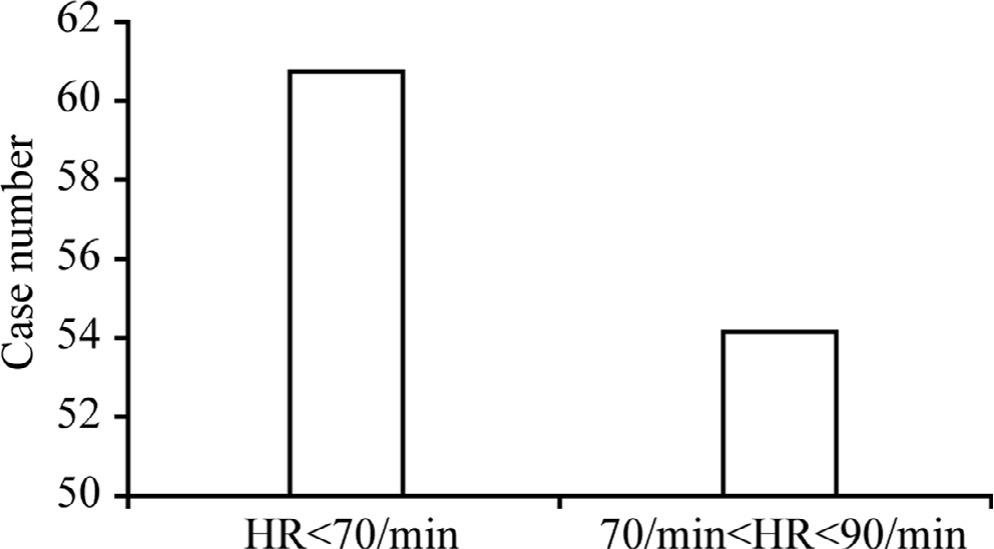
Comparison of case proportion of Score 1 image quality between HR < 70/min and 70 ≤ HR < 90/min.

Excellent and good image quality of RCA scorings, are described on Score 1 and Score 2, respectively. To the images belongs to Score 1, there is no statistical difference on the case number proportions of prospective ECG‐gated method using 320‐ row CT scan (Toshiba) between HR < 70/min and 70 ≤ HR < 90/min (*P* value at 0.622, *P *> 0.05). There is also no statistical difference on the case number proportions using 2nd generation (128‐ slice) dual source CT scan (Siemens) between HR < 70/min and 70 ≤ HR < 90/min (*P* value at 0.431, *P *> 0.05). However, there is higher proportion of case number that belongs to Score 1 with HR < 70/min (Fig. 2; Table 2).

**Table 2:** Right coronary artery image quality score.

Score (n, %)	Prospective gated method of 320 row CT scan (Toshiba)		Prospective gated method of 2nd generation (128‐slice)dual source CT scan (Siemens)	
	HR < 70/min	70/min < HR < 90/min	HR < 70/min	70/min < HR < 90/min
1	35 (59.32)	31 (50.82)	32 (67.75)	26 (59.09)
2	24 (40.68)	29 (47.54)	17 (33.33)	17 (38.64)
3	0	1 (1.64)	2 (3.92)	1 (2.27)
4	0	0	0	0
*P* value	0.622		0.431	

For images that belong to Score 1 plus Score 2, there is no statistical difference on the case proportions using prospective ECG‐gated method of 320‐ row CT scan (Toshiba) between HR < 70/min and 70 ≤ HR < 90/min (*P* value at 0.927, *P*> 0.05). And there is also no statistical difference on the case proportions using 2nd generation dual source CT scan (Siemens) between HR < 70/min and 70 ≤ HR < 90/min (*P* value at 0.532, *P *> 0.05).

To the HR group of <70/min, there is no statistical difference of case number proportions that belongs to Score 1 and Score 1 plus Score 2 using prospective ECG‐gated method between 320‐row CT (Toshiba) and 2nd generation (128‐slice) dual source CT (Siemens) (*P* values are 0.714 and 0.336, respectively, *P *> 0.05).

**Fig. 3:**
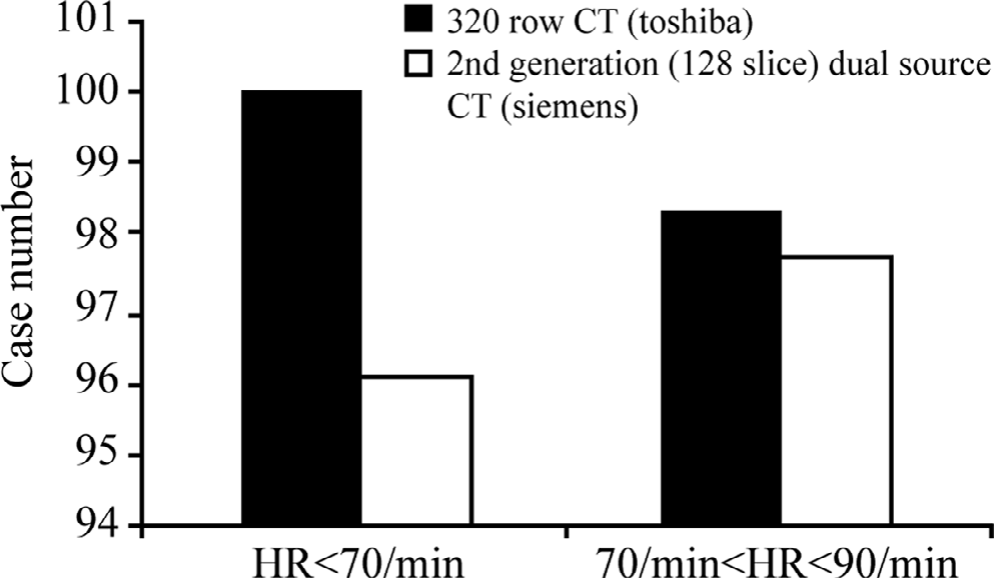
Comparison of case proportion on Score 1 plus Score 2 image quality between HR < 70/min and 70 ≤ HR < 90/min, and matching prospective ECG gated method between 320‐row computed tomography (CT) scan (Toshiba) and 2nd generation (128‐slice) dual source CT scan (Siemens).

To the HR group of 70 ≤ HR < 90/min, there is no statistical difference of case number proportions that belongs to Score 1 and Score 1 plus Score 2 using prospective ECG‐gated method between 320‐row CT (Toshiba) and 2nd generation dual source CT (Siemens) (*P* values are 0.508 and 0.094, respectively, *P *> 0.05). However, there is higher case number that belongs to Score 1 and Score 2 using 320‐row CT (Toshiba) (Fig. 3).

### 3.B Effective CT radiation dose

To the groups of HR < 70/min and 70 ≤ HR ≤ 90/min, the effective radiation dose of prospective ECG‐gated method between 320 row CT (Toshiba) and 2nd generation (128‐slice) row dual source CT are shown in Table 3. In Table 4, the effective radiation dose between prospective and retrospective ECG‐gated method of 2nd generation (128‐slice) row dual source CT are shown.

**Table 3:** Effective radiation dose of the two computed tomography (CT) devices on two different HR groups.

	CT device	N	Effective radiation dose (mSv)	*P* value
HR < 70	Prospective gated method of 320 row CT scan (Toshiba)	59	3.3557 + 2.35413	<0.05
	Prospective gated method of 2nd generation (128‐slice) dual source CT scan (Siemens)	51	2.0865 + 0.4284	
HR ≥ 70 ≤ 90	Prospective gated method of 320 row CT scan (Toshiba)	61	4.1909 + 2.60384	<0.05
	Prospective gated method of 2nd generation (128‐slice) dual source CT scan (Siemens)	44	1.7581 + 0.4341	

**Table 4:** Effective radiation dose of the two methods [2nd generation (128‐slice) dual source CT scan (Siemens) on two different HR groups.

	Method	N	Effective radiation dose (mSv)	P value
HR < 70	Prospective gated method	51	2.0865 + 0.4284	<0.001
	retrospective gated method	50	14.5810 + 7.5422	
HR ≥ 70 ≤ 90	Prospective gated method	44	1.7581 + 0.4341	<0.001
	Retrospective gated method	50	10.0727 + 7.7637	

The result indicates effective radiation dose of 2nd generation (128‐slice) row dual source CT on both HR groups are lower than 320 row CT (Toshiba) and effective radiation dose of prospective ECG‐gated method on both HR groups are lower than retrospective ECG‐gated method of 2nd generation (128‐slice) row dual source CT. There are significant statistical differences (*P* < 0.05, *P* < 0.01) between the two CT devices and two methods in these two HR groups.

**Fig. 4:**
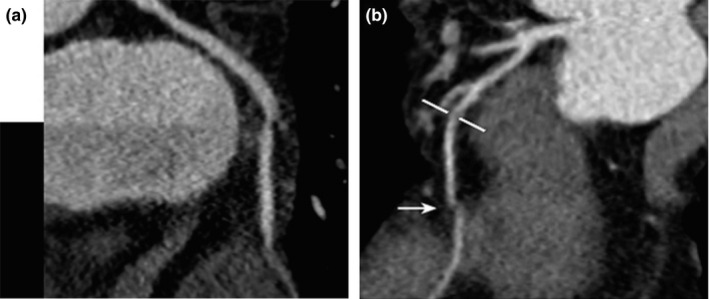
(a) Step artifact caused by step‐and‐shoot acquisition, shown by the black and white discrimination. (b) Heart rate variation that producing the stair‐step artifacts (white arrow) that mimicking stenosis, and white bars highlight true stenosis.

Once the rapid and instable HR is beyond the limitation, the combination of non‐synchronicity and displacement of coronary artery, as well as out of phase cross‐sectional scanning module, may cause step artifacts [Fig. 4(a)]. Furthermore, it progresses to stair‐step artifacts [Fig. 4(b)].

## 4 DISCUSSION

### 4.A Effect of heat rate on the image quality of RCA

As the progression of CT technology on short scan time and higher spatial resolution, there is lesser degree of correlation between the image quality of coronary artery to heart rate.^14^, ^15^, ^16^ Our result shows no statistically significant difference between the two different HR groups on excellent and good image quality of RCA, as shown in Table 3. The case number proportion of excellent and good RCA image on low HR group is better than the high HR group. And thus, we still advise an appropriate dose of beta blocker for HR control, before the CCTA examination. About the technological advantages of these two CT devices, the 2nd generation (128‐slice) dual‐source CT (Siemens) is composed by two sets of independently operating X‐ray tubes and 64 row detectors, it finishes a cardiac scanning at 280 ms.^17^ Using single sector reconstruction, the temporal resolution is 75 ms. According to Flohr other studies,^18^ once the temporal resolution is below 83 ms by using single sector reconstruction, image quality is not related to by heart rate. On the other hand, 320 row CT scan (Toshiba) has wider coverage scanning at 275 ms/cycle. It allows to obtain an entire cardiac volume by non‐helical scanning, without necessary table movement. For different heart rates, 320 row CT scan (Toshiba) can automatically select single or multiple R‐R interval exposure. It also combines the technology of multi‐sector reconstruction, effectively to improve the temporal resolution, and then ensure a high quality image outcome.^19^ To the cases with HR < 70, 320 row CT scanner (Toshiba) can successfully complete a whole cardiac volume within one cardiac cycle. And to the cases with 70 ≤ HR < 90/min, it may need two cardiac cycles.

### 4.B Comparison of RCA image quality on the same group of different range of heart rates

Our result shows both heart rate groups of <70/min and ≥70 HR ≤ 90/min have no significant difference between the two scanners by the summation case numbers of RCA image score 1 and score 1 + 2. The case number proportion of excellent and fine image quality of the two CT scanners are 99.17% and 96.84%, respectively. As aforementioned, both of the image results of the two CT devices have met the clinical requirement, but 320 row CT (Toshiba) has a higher image quality performance.

Compared to 2nd generation 128 dual source CT (Siemens), 320 row CT (Toshiba) has a wider scanning detector with coverage for 16 cm length on table in a single gantry rotation. For candidates with lower heart rate, one single cardiac cycle using single sector reconstruction is enough to finish a whole cardiac volume scanning. It also remarkably reduces the case number proportion with step or stair‐step artifacts caused by R‐R interval inconsistencies during reconstruction. In conditions of high level heart rate and arrhythmia, it needs multiple heart cycle scan for multi‐sector reconstruction to improve the temporal resolution. Through the ECG editing function, more than 99% case excellent and good RCA image quality (Score1 + 2) got. But practically, candidates with higher and variable HR cause inconsistent R‐R interval, the data matching of multi‐sector reconstruction is also inconsistent. Even though images are received ECG editing, there is some blurred artifacts. A case example who had instable HR at about 89/min of using 320 row CT scanner (Toshiba), was belongs to RCA image quality at Score 3, the image was free from stair‐step artifacts. However, there was some blurred artifacts due to relatively low temporal resolution. In contrast to retrospective ECG gated, candidate selection for prospective ECG‐gated method on 2nd generation (128‐slice) dual source CT (Siemens) is highly affected by instable and rapid heart rate. with compensatory function the scanning program automatically to skip or repeat to the next cardiac cycle scanning. For prospective ECG‐gated method on 2nd generation (128‐slice) dual source CT (Siemens), candidates could be allowed for scanning when the HR variation is smaller than 10/min.

### 4.C Comparison on effective radiation dose of CT scanning

Radiation damage of CCTA was considered since the study report published by Redberg et al.^20^ How to keep a balance of reducing effective radiation dose and maintaining image quality of coronary CT scanning is a hot research topic at present.^21^ In our study, the effective radiation dose of both heart rate of HR < 70/min and 70 ≤ HR ≤ 90/min when using prospective ECG‐gated method on 2nd generation (128‐slice) row dual source CT (Siemens) is lower than the same method on 320 row CT (Toshiba). To explain the higher effective radiation dose on 320‐ row CT (Toshiba), we found the Toshiba manufacturer presets its bolus tracking on threshold value of contrast medium on ascending aorta and the corresponding threshold value is over 150HU with scanning cycle at 1/s.

The purpose of bolus tracking is to monitor and guarantee an appropriate amount of contrast concentration that can pass through the target vessel matching the candidates' heart rates and left ventricular outputs. Pratically, the pre‐scanning threshold value of bolus tracking focus on ascending aorta can be as high as 150 to 300 HU. In the future, we may consider to have an alternative method to replace this bolus tracing, so as to reduce the additional effective radiation dose on 320 row CT scanner (Toshiba). In practical, the criteria of patients selected for prospective‐gated method for 2nd generation (128‐slice) dual source CT (Siemens) is rather stringent because of the high failure rate on patients with high level HR variation or even atrial fibrillation. Instead, CCTA on these kinds of candidates were then completed using retrospective ECG‐gated method. Thus, prospective ECG gated method using 2nd generation (128‐slice) dual source CT (Siemens) cannot be completely adopted, although it has the advantage of low effective radiation dose.

On the contrary, the application of prospective ECG‐gated method on 320 row CT (Toshiba) is not limited on these factors with high successful rate. Clinically, we had never performed retrospective ECG‐gated method for CCTA using 320 row CT (Toshiba).

This study has some limitations, that include: (a) image reconstruction on RCA image quality is manually selected and subjectively optimized the best phase (%) of cardiac cycle; (b) although most of the candidates with heart rate over 90/min were successful adjusted by 25–50 mg beta‐blocker,^22^ there were still some cases excluded in this study, due to uncontrolled high level and variable HR; (c) to the same reason, some cases were excluded when they were assigned to use retrospective ECG‐gated method on 2nd generation dual source CT (Siemens). And thus, this study is not in true randomized sampling; (d) some cases were excluded due to serious calcified plaques depositions and too small of coronary diameter, it may produce biased statistical results; (e) relatively low sample number; (f) The image quality also depends on the post‐processing level of the technicians. Although all the staff involved in the post‐processing work have more than 3 yr of rich working experience, the consistency of the post‐processing level cannot be guaranteed, thus it may have certain influence on the post‐processing image score. (g) There are 100 cases of retrospective gating on Siemens scanner, which are not included in this study.

## 5 CONCLUSION

For the patients with high level heart rate variation, both prospective ECG‐gated method of 320 row CT scan (Toshiba) and 2nd generation dual source CT scan (Siemens) were basically satisfactory for clinical diagnosis on the image quality of RCA. There is lower effective radiation dose on prospective ECG‐gated method using 2nd generation dual source CT scan (Siemens). There was low failure rate on the prospective ECG‐gated method using 320 row CT scanner (Toshiba) even on poor control heart rate candidates, on the contrary, using 2nd generation dual source CT scanner (Siemens) may need to adopt the retrospective ECG‐gated method when prospective ECG‐gated method is failure on rapid and instable HR group, while retrospective method is known to provide higher effective radiation dose. In our opinion, if patients have variable, irregular or fast heart rate, CT could lead to higher successful rate and more safety in radiation dose exposure.

## CONFLICT OF INTEREST

None.
